# The rationale for and use of assessment frameworks: improving assessment and reporting quality in medical education

**DOI:** 10.1007/s40037-015-0182-z

**Published:** 2015-05-12

**Authors:** Jacob Pearce, Daniel Edwards, Julian Fraillon, Hamish Coates, Benedict J. Canny, David Wilkinson

**Affiliations:** 1 Australian Council for Educational Research, Private bag 55, 3124 Camberwell, VIC Australia; 2 University of Melbourne, Melbourne, Australia; 3 Monash University, Melbourne, Australia; 4 Macquarie University, Sydney, Australia

**Keywords:** Assessment framework, Assessment, Reporting, Quality

## Abstract

An assessment framework provides a structured conceptual map of the learning outcomes of a programme of study along with details of how achievement of the outcomes can be measured. The rationale for using frameworks to underpin the targeting of essential content components is especially relevant for the medical education community. Frameworks have the capacity to improve validity and reliability in assessment, allowing test developers to more easily create robust assessment instruments. The framework used by the Australian Medical Assessment Collaboration (AMAC) is an interesting and relevant case study for the international community as it draws and builds on established processes in higher education assessment. The AMAC experience offers an insight into important considerations for designing assessment frameworks and implementing frameworks in differing contexts. There are lessons which have the potential to improve assessment and reporting practice and quality in not only medical education, but in other domains of assessment. Prior to implementing any programme of assessment, the framework considerations outlined here will hopefully improve the quality of assessment and reporting practice by making implicit assumptions explicit, and allowing more critical reflection and evaluation throughout assessment processes.

## Introduction

This paper discusses the rationale for assessment frameworks and the use of a particular framework in the field of medical education. Firstly, assessment frameworks are defined in relation to construct validity and programmes of assessment. The rationale for using assessment frameworks in general is then discussed. This is followed by outlining the use of an assessment framework as part of the Australian Medical Assessment Collaboration (AMAC) [1]. This collaborative endeavour between an independent research organization and 16 of the 19 medical schools in Australia and New Zealand involved assessing final year medical students completing clinical training. The framework is presented, and important considerations for future work emerge. We conclude by presenting a series of notable complexities associated with using the framework. Considerations and challenges relating to future framework development and deployment are presented, with an overarching focus on improving assessment and reporting quality.

## What is an assessment framework?

Assessment frameworks provide a structured conceptual map of the learning outcomes of a programme of study. Where curriculum frameworks detail what is to be taught, assessment frameworks detail what is to be assessed as evidence of learning described by the requisite curriculum content. Built into an assessment framework are assessment concepts (and their definitions), along with theoretical assumptions that allow others to relate to the framework and potentially adapt it to other domains of assessment. Further, an assessment framework details how an assessment is to be operationalized. It combines theory and practice, and explains both the ‘what’ and the ‘how’ [2].

The process of assessment can be conceived as a measurement of the expression of a construct by individual candidates. A framework articulates the construct(s) to be measured, and the links between the construct(s) and the design and content of the instrument(s). A framework includes blueprinting of the components of the construct that are to be covered in the assessment instrument. The validity of both the instrument and the framework can be evaluated by monitoring the mapping of tasks to the construct components. Here, it is worth stating that we subscribe to the theoretical framework advocated by Kane, whereby the validity of conclusions drawn regarding the measurement of candidate attributes is treated on the basis of a series of inferences from assessment results [3–5]. Thus, assessment effectively means measuring achievement against a construct.

Methodological, technical and pragmatic considerations form part of the framework document, along with considerations of what is appropriate and feasible to assess. A framework functions as a reference system against which to evaluate whether individual tasks target the specified learning outcomes and collectively represent the desired coverage of assessment content. In an assessment framework the purposes of assessment can be articulated in greater clarity and theoretical assumptions and desired outcomes can be made explicit [5, 6].

In the medical education community there has been much discussion of the methodologies involved in assessment, and criticism levelled at the notion of construct validity [7–10]. However, scant attention has been paid to the rationale for assessment frameworks *per se*. Traditionally, curricula are well blueprinted, and are implemented through careful programme planning and tailored teaching and learning resources. Assessment, however, is often afforded fewer planning resources and (at least in Australia) left largely in the hands of academic staff as they deliver courses with, in many cases, relatively little training or support in assessment science. We now turn to the rationale for assessment frameworks to complement other educational programme resources.

## The rationale for assessment frameworks in medical education

The rationale for using frameworks to underpin the targeting of essential content components for assessment is especially relevant in the current medical education climate [9]. The medical education community has shifted its focus away from developing ‘holy grail’ assessment instruments that rely on assessing idiosyncratically defined essential pieces of content for the separate constructs that make up medical competence [5], towards separate instruments for different purposes. Schuwirth and Van der Vleuten assert that the *content* of an assessment is far more important than its *format* [5, 11, 12]. Content must be of high quality, and appropriate means for defining and evaluating quality must be agreed upon [13].

Frameworks have the capacity to improve both validity and reliability in assessment, and allow test developers to more easily create robust assessment instruments. This is achieved particularly by improving the clarity of articulation of the purpose of the assessment tasks; providing a reference point during assessment development; and ensuring there is clear mapping to components and dimensions of the framework when undertaking psychometric analyses. Using frameworks, specific competencies can be targeted through different assessment instruments built according to agreed-upon framework components. As Amin states, ‘we need to articulate the purpose of the particular assessment with the greatest possible clarity in a manner that goes beyond its simple categorization as summative or formative. We must ask repeatedly what the real purpose of assessment is and be certain of its explicit, as well as implicit, agenda’ [6]. A well-articulated assessment framework fulfils this purpose.

Having a common assessment framework is one way to support consistency of assessment within and across institutions. In a field such as medical education, which demands high standards and comprises consistent scientific content, the use of a framework as the foundation for building assessments is feasible and arguably desirable. As an explicit articulation of agreed definitions and standards, a framework acts as a consistent point of reference for a community. The existence of a framework encourages the critical, reflective development of instruments, increases accountability and can reduce bias in assessment practice. The quality of assessment can also be improved and innovative reporting pathways can be developed [14, 15].

However, until recently, medical schools in much of the world (aside from Progress Testing in the Netherlands perhaps [16]) have largely undertaken assessment of student performance in isolation from each other [17]. If assessment frameworks are deployed in the context of cross-institutional collaborative assessment programmes, they have the potential to enhance the capacity of medical educators in enabling their students to develop the necessary skills, knowledge, attitudes and behaviours to fulfil their future roles [18].

There is also a wider and increasing focus in both professional and academic literature on the importance of having well-defined assessment frameworks to measure the learning outcomes of students [19–22]. Interest in implementing international medical assessments is also gaining pace [23–26]. As part of the Organization for Economic Co-operative and Developments (OECD) Assessment of Higher Education Learning Outcomes (AHELO), for instance, tests were developed and applied in 17 countries to measure ‘Engineering Proficiency’, ‘Economics Proficiency’ and competence in ‘Generic Skills’ [27]. Instrument development in each of these strands in the AHELO Feasibility Study was guided by assessment frameworks [28–30]. How the domains were represented and organized in the frameworks informed the assessment design and, ultimately, the evidence about student proficiencies that could be collected and reported. These processes and technical procedures evolved through multi-national assessments of school-aged students such as in the Programme for International Student Assessment (PISA) across 70 countries at the secondary school level [31], the Trends in International Mathematics and Science Study (TIMSS), and many other international studies. In this regard, medical education has much to gain from each of these studies, which use assessment frameworks effectively to improve both assessment and reporting.

## The use of an assessment framework in medical education

### The Australian Medical Assessment Collaboration (AMAC)

While the AMAC collaboration formed in Australia, the example it provides is relevant across educational systems and disciplines. The project, funded by the Australian Learning and Teaching Council (now the Office of Learning and Teaching), was initiated in 2011 as a partnership between the University of Queensland, Monash University and the Australian Council for Educational Research (ACER). The next phase, completed in 2014, involved ACER partnering with 16 of the 19 medical schools in Australia and New Zealand.

The AMAC project included scoping work, sector-wide consultation, framework development, the collaborative review and revision of assessment tasks, and pilot testing of the tasks using data provided by over 2000 final year medical students in 11 different medical schools across 20 different student cohorts in both formative and summative settings. The outputs included three comprehensive ‘manuals’ outlining considerations for future assessment collaborations in any discipline, in terms of assessment quality, implementation, and governance and dissemination [13, 32–34].

AMAC is a relevant case study for the international medical education community as it draws and builds on established processes in higher education assessment with the potential to generate comparable data on student learning outcomes across institutions and countries [35, 36]. AMAC responded to a perceived need for evidence-based measures on which to establish graduate capability, measure success, and facilitate continuous improvement in medical education [37]. It provided conceptual and operational foundations for the ongoing development and implementation of assessment instruments designed to offer medical schools a sustainable and robust resource to support the monitoring of learning outcomes and to use these data to evaluate their own learning and teaching programmes.

### The framework context

For AMAC, an assessment framework was developed and used as the basis of instrument development [1]. Through a process of consultation and collaboration, it became clear that in the Australian medical community there was concern that consensus on assessment frameworks is difficult to achieve. There are many conflicts and struggles that are encountered by stakeholders, which may cause some to think that frameworks are unscientific―the precise articulation in any framework will most certainly be influenced and informed by individuals, institutions, governments, and other stakeholders involved in its development, at any particular point in history.

This concern was overcome by the recognition that a framework is only ever one possible way of describing the content and structure, the multifarious possibilities and complexities of a specific domain at a given point in time. Developing a fixed or unchanging assessment framework for medical education would neither be feasible nor desirable. Regardless of its detail and structure, an assessment framework offers the opportunity to establish a common language and set of understandings of the assessment outcomes in a specific educational context. This provides the ancillary benefits associated with having a community of people being involved in continuous improvement.

Consensus on the framework was achieved through iterative, collaboration design, development and review. Doctors, clinical teachers, medical education experts and assessment specialists were all involved in this process. When there was disagreement, it related to specific categorizations, rather than fundamental criticisms. This was perhaps due to the fact that all involved in the AMAC project subscribed to the same value proposition―that an assessment framework was an essential component of the project. Having a dedicated group of people taking the lead on framework development at ACER ensured that individual and collective concerns were addressed promptly, and re-circulated for comment.

The AMAC assessment framework was informed by national and international assessment frameworks and curriculum documents [19, 20, 28–30, 38–41]. A provisional framework was drafted by ACER and project partners in mid 2011 and presented to participants at an engagement forum involving all medical schools and key stakeholders from Australia and New Zealand. Following the forum, the draft framework was revised. Further consultation on the framework was undertaken in late 2011 in workshops with clinical academic staff representing a range of specializations, leading to several minor revisions. In practice, the framework is considered to be a ‘living document’ opening the possibility for further review and revision. The result is a comprehensive assessment framework that builds on and aims to improve what is presently available in the public sphere. The AMAC framework adds value to the assessment literature and will inform future developments.

### The framework architecture

There are many sensible and defensible ways that a domain can be divided. With this in mind, the most important parameter for the AMAC project team was that the framework could comprehensively accommodate all requisite components. If the description of the domain were to be organized in a way that certain aspects of medical education-learning outcomes, competencies, specialities, etc.-could not fit, then the framework was considered flawed. The lists in the sub-domains of the framework were purposely detailed. The reason is that with a high level of granularity, there is greater flexibility in how assessments are designed. Once a decision is made regarding what is to be assessed, the concepts and categories in the sub-domains may be aggregated or collapsed for a purpose, but they do not have to be.

Following is an outline of the structure and contents of the assessment framework. The areas for possible assessment were divided into two domains: *Content* and *Process*. The two domains function together so that any given task can be mapped to the content it addresses and the application of that content in either cognitive or practical contexts. A third dimension is the clinical context, which situates the expression of proficiency.

**Fig. 1:**
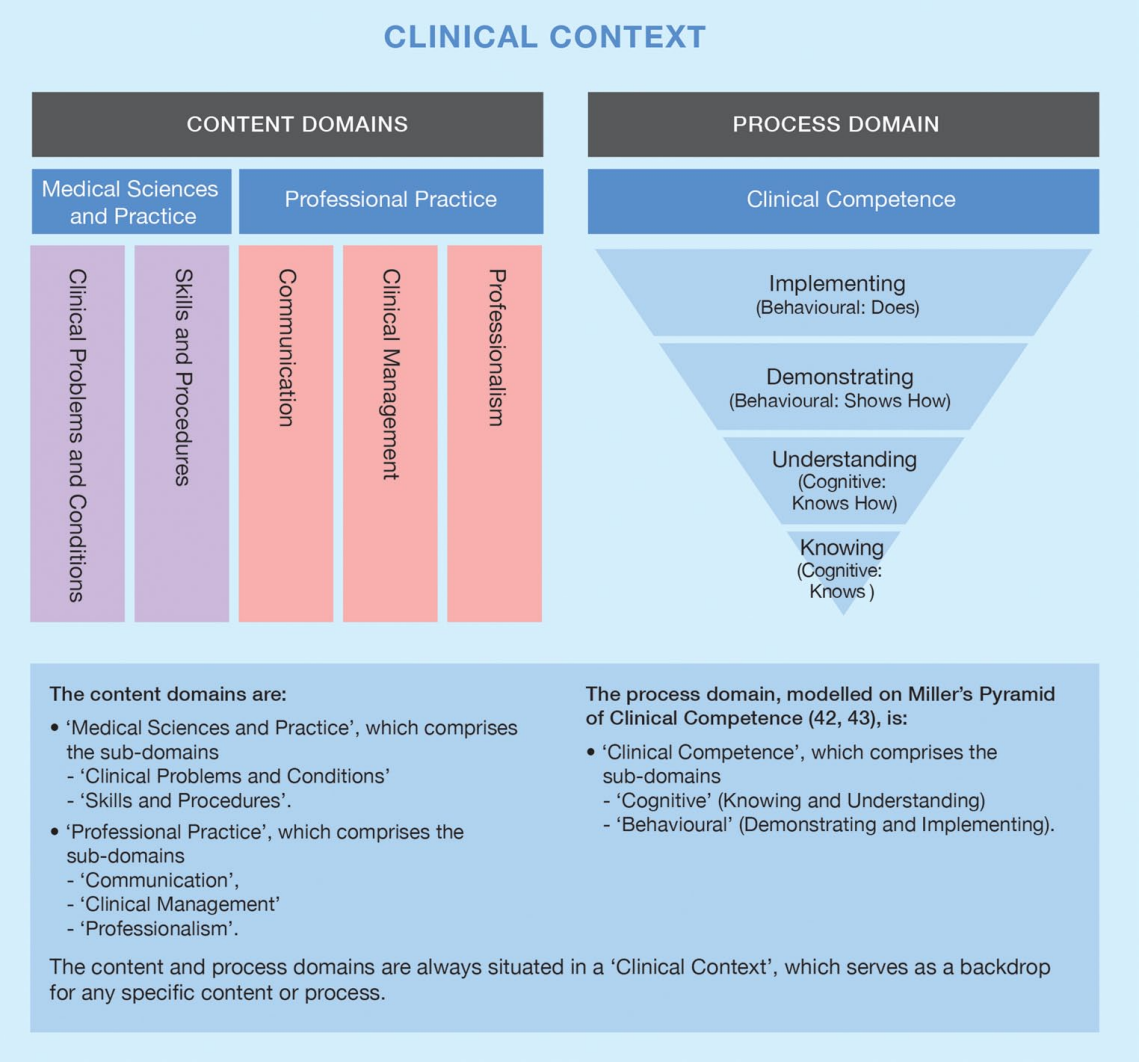
Framework components

Figure 1 illustrates that the content domains are coupled with the process domain. The vertical bar for each content sub-domain demonstrates that each sub-domain can be assessed through a cognitive or behavioural process, and always in a clinical context. All five of the content sub-domains can be mapped to both of the process sub-domains. The intended implication of Fig. 1 is that the practitioner’s capacity broadens rather than narrows with increasing proficiency. For this reason, while the hierarchy of competencies based on Miller’s pyramid has been maintained, the pyramid shape has been inverted (Fig. 1).

Once the domains were described and agreed upon, detailed sub-domains were articulated. For example, the Clinical Problems and Conditions sub-domain has four constituent classification categories [1]:

1. The *system* involved. This list is of all the major systems in the human body, including respiratory, circulatory, nervous, and others;
2. The *medical speciality*. This list is based on the Australian Recognized Medical Specialities, including haematology, urology, cardiology, and others;
3. The *medical context*. This list comprises places and contexts where medical issues arise, including primary care, emergency department, and others; and
4. The *demographic*. This list comprises groups of individuals, including adult health, paediatrics, aged care, rural, and others.

The *Clinical Context* dimension contextualizes both the content and process involved in a task, and situates the expression of proficiency. It includes concepts such as making a diagnosis, decision making, medical testings, and others.

The framework provided a consistent reference point for classifying the assessment properties of assessment tasks. This allowed for the development of a profile of the content, process and contextual coverage provided by sets of tasks. New assessment tasks could be generated with specific reference to each of the three dimensions. Tasks could be developed to target one of the content domains, one of the process domains, and be situated in one of the clinical contexts. In addition, existing assessment tasks from other sources were classified according to these three dimensions. If one of the dimensions was missing, it was clear in the process of categorization but regardless, the assessment framework provided a coherent foundation for ensuring that an assessment was complete.

### An application of the AMAC framework

The pilot AMAC instrument, developed with reference to the assessment framework, was administered from 2011–2013. The instrument included 120 tasks, and assessed the Clinical Problems and Conditions sub-domain of the Medical Sciences and Practice content domain, and the Cognitive process sub-domain (knowledge and understanding) of the framework, always in a Clinical Context. The Skills and Procedures sub-domain and the Behavioural process sub-domain were not assessed in the AMAC pilot, but parts of the Professional Practice content domain were.

For this application of the framework, only multiple choice questions (MCQs) were deployed due to project constraints. More details of the development of the assessment instrument are available elsewhere [32, 36, 44]. Existing tasks were modified in assessment workshops and mapped to the framework [13]. Decisions were made regarding the balance of content, which is detailed in the framework document [1]. Where there were gaps in the framework components that were desired in the assessment instrument, certain types of tasks were requested or developed. This instrument was seen as just one application of the framework for a specific purpose-other instantiations balancing content and types of assessment in different ways could equally be undertaken using the same framework. The framework discusses different types of assessment and how these can be categorized [1].

The MCQs were mapped to the four categories under the Clinical Problems and Conditions sub-domain, the relevant aspect of Professional Practice, and their Clinical Context. For example, a task that presented a short vignette about a woman presenting with easy bruising and showing multiple petechial haemorrhages (amongst other relevant contextual information) required candidates to identify which laboratory investigation would most likely lead to a diagnosis. This item was classified as ‘Understanding’ for Process Domain, ‘Circulatory’ for Medical System, ‘Haematology’ for Medical Speciality, ‘Primary Care (includes general practitioner)’ for Clinical Context, ‘Adult Health’ for Demographic, ‘Medical Testing’ for Clinical Context, and ‘Patient Assessment’ for Professional Practice.

Although some classifications across categories were more strongly linked than others (for instance, an item will undoubtedly be categorized as ‘endocrine’ for Medical System, along with the ‘endocrinology’ Medical Speciality), demarcating aspects of Clinical Problems and Conditions across these four categories ensured that no information was lost in classifying tasks. This process was valuable in that it allowed a distinction between the theoretical and practical aspects of medical content to be encapsulated. Medical specialities often pertain to what individuals do, whereas medical systems pertain to how ideas may be organized. The medical context pertains to localized conditions, whereas the demographic pertains to the relevant individuals.

Although the aim of the classification system was to allow for unique mapping of assessment tasks across content, some redundancy in classifications was an acceptable outcome of having the system reflect the interconnectedness of the constituent elements of medical practice, whilst ensuring complete coverage of the domain. The aim was to capture as many concepts as possible in classifying assessment tasks. This process is similar to a ‘tagging’ process utilized in many online classification systems [45, 46] and should be considered analogously. Defining the sub-domains in this way allowed test developers to show a clear map of the assessment tasks in the instrument. It also allowed assessors to dictate their desired emphasis in any assessment instrument. Participants in the AMAC review workshops had no trouble tagging other types of assessment tasks as well, although these were not included in the final pilot assessment instrument.

The project team noted that in other national and international assessments, conflation between these distinct aspects of this sub-domain frequently occurs. For instance, the International Foundations of Medicine (IFOM) Clinical Science Examination, administered by the National Board of Medical Examiners (NBME), states that ‘organ system’ comprises over 95 % of their examination, but the list under this heading includes ‘systems’, ‘diseases’ and ‘disorders’ along with implicit demographics in the categories [47].

## Considerations for future framework development and use

The AMAC experience offers insight into important considerations for designing assessment frameworks and implementing frameworks in differing contexts of assessment programmes. It is perhaps worth flagging the sheer amount of effort that went into this exercise. Others have examined the implementation side of the AMAC project, and the practical benefits associated with improving assessment processes [32, 35, 36]. We argue that the AMAC case achieved many of the proposed benefits of utilizing an assessment framework: improved validity and reliability in assessment; better quality reporting; and improved clarity of articulation of the purpose of the assessment tasks [32, 44].

The nature of the AMAC collaboration was that existing medical schools, with existing and different assessment regimens and curriculum frameworks, came together to see if they could establish a common assessment framework. Our results and observations suggest that this can be achieved, but perhaps only within contexts where a high degree of agreement exists regarding the nature of curriculum content. It is fortunate that in a professional field, such as medicine, this generally occurs. As ‘ideal’ curriculum development involves incorporation of an assessment framework from the beginning of planning, we believe that a framework, such as ours, could be applied in the early stages of curriculum development to address generally agreed curriculum outcomes.

The lessons from the AMAC framework experience are generalizable beyond the medical education field, and the Australasian context. The use of the assessment framework was complex and illuminating, and has the potential to improve assessment and reporting practice and quality in not only medical education, but in other domains of assessment. Complexities in medical curricula were coupled with professional complexities when multiple stakeholder perspectives were synthesized to establish a complete, coherent and consistent framework. Several challenges and developments are worth highlighting.

### Generality versus specificity

There seem to be two possible broad methodologies for developing an assessment framework: one based on generality, the other specificity. A framework guided by generality would see more aggregation of traditional distinctions in the medical discipline. A framework which follows this route is taken by the Australian Medical Council, where systems, regions and disciplines are treated collectively [39]. The alternative, adopted by AMAC, was to present a framework with increased levels of granularity. In this way, a large component of the framework consists of systems, specialities, contexts and demographics, which were *not* assessed. However, in doing so, their assessment possibility is recognized.

### Framework completeness

One issue in reviewing existing frameworks was the problem of specific elements not fitting into certain categories-the question of ‘completeness’. There is a plethora of ways of dividing a discipline, and multifarious possible framework incarnations. Appreciating this fact, it is ideal to attempt completeness in designing assessment frameworks. Even if certain elements are not assessed in particular assessment instruments, the roadmap of what it is possible to assess must be broad, complete and internally consistent. The framework should comprehensively accommodate all requisite components.

### Measuring competencies

The majority of higher-level competencies were not targeted in the AMAC case study and further work is required to ensure a robust assessment framework at this level. It is hoped that future work will make inroads in these areas. Fernandez et al. state ‘much more nuanced descriptors of clinical performance are essential if we are to understand the richness of clinical competency’ [48]. Further developing these areas of the AMAC framework has the capacity to measure more abstract competencies in a concrete and grounded way.

### Mapping assessment tasks

The importance and relevance of mapping in item banks was underscored throughout the AMAC project. Assessment tasks were mapped to the framework with the aim of ensuring a one-to-one mapping across all elements in the selected domains for assessment. The process began with the idea of ‘tagging’, for each MCQ, before the list (and the task itself) was modified to ensure that it mapped completely. Tasks could be stored in ‘item banks’ for retrieval at a later date. This aids the instrument development process and allows for careful instrument development for specific purposes.

An important caveat to be borne in mind in mapping assessment tasks is that while an individual task may be mapped against multiple domains, it does not necessarily address each of the domains with equal relevance, and an over-reliance on the mapping may lead to false conclusions about the blueprint of an assessment or the competence of an individual. Mapping is probably best suited to item banking purposes, and to provide a guide to blueprinting. No automated process can circumvent the academic responsibility of ensuring the validity of an assessment task presented to students.

### Reporting potential

An assessment framework built upon the ideals of completeness and specificity, with consistent and distinct demarcation of categories, increases the potential in the reporting realm. A more detailed breakdown of student performance can be reported and compared according to the framework architecture. A medical school may be able to pinpoint weaknesses amongst a cohort of students, such as a weakness in ‘decision making’ or the ‘immune system’. Further, instruments can be designed to target specific areas, which educators believe may be lacking in a programme, such as the ‘nervous system’ in the ‘Emergency Department’. More on the reporting potential in AMAC is covered elsewhere and example student and institution reports are given [32, 34].

### Benchmarking potential

The question of reporting relates to the construction of the scales that are reported. An achievement map can be developed whereby progress can be followed on certain sub-scales, which are constituents of medical competency. The AMAC framework implicitly pointed to different levels of achievement across the process and content sub-domains. Although it is too soon to construct a scale (and sub-scales) for medical competence based on the AMAC framework, the framework offers this possibility. The power here lies in providing institutions with data on the level of attainment of learning outcomes of its students according to clearly defined scales. This enables benchmarking across institutions and, potentially, across countries [32, 34, 35].

### Social issues

Finally, we bring attention to an interesting sociological complexity of assessment frameworks. If a framework does not exist, and an instrument is developed without reference to one, the community seems less likely to argue about the make-up of the assessment instrument. However, when a framework exists, it is thrown into the public domain and susceptible to critique. This is the philosophical tension-frameworks are needed to ensure robust assessment, but once they exist, they encourage critical reflection. This critical reflection is advantageous, but should result in the continuous improvement of frameworks, rather than their rejection.

## Conclusion

The lessons to emerge from the AMAC experience are useful not only for the international medical education community, but extend to other disciplines working towards the design, development or deployment of assessment frameworks. Prior to implementing any programme of assessment, the framework considerations outlined here will hopefully improve the quality of assessment and reporting practice by making implicit assumptions explicit, and allowing more critical reflection and evaluation throughout assessment processes. The hope is that these considerations improve both the quality of tasks (and associated concepts such as content and construct validity for particular programmes of assessment), and the quality of reporting that can inform learning and teaching.

## Essentials

- An assessment framework provides a conceptual map of the learning outcomes of a programme of study along with considerations for how assessment is to be operationalized.
- Frameworks have the capacity to improve validity and reliability in assessment, allowing test developers to more easily create robust assessment instruments.
- The framework used by in AMAC is an interesting and relevant case study for the international community as it draws and builds on established processes in graduate assessment.
- Important considerations emerge for designing and implementing frameworks in differing contexts. These have the potential to improve assessment and reporting practice and quality in not only medical education, but in other domains of assessment.
- The framework considerations outlined here allow more critical reflection and evaluation throughout assessment processes.
